# Sex-specific alterations in functional connectivity and network topology in patients with degenerative cervical myelopathy

**DOI:** 10.1038/s41598-024-67084-9

**Published:** 2024-07-11

**Authors:** Talia C. Oughourlian, Shan Rizvi, Chencai Wang, Alex Kostiuk, Noriko Salamon, Langston T. Holly, Benjamin M. Ellingson

**Affiliations:** 1 UCLA Brain Tumor Imaging Laboratory (BTIL), Center for Computer Vision and Imaging Biomarkers, David Geffen School of Medicine, University of California Los Angeles, Los Angeles, CA USA; 2 Department of Radiological Sciences, David Geffen School of Medicine, University of California Los Angeles, 924 Westwood Blvd, Suite 615, Los Angeles, CA 90024 USA; 3 Neuroscience Interdepartmental Graduate Program, David Geffen School of Medicine, University of California Los Angeles, Los Angeles, CA USA; 4 Neuroscience Undergraduate Interdepartmental Program, College of Life Sciences, University of California Los Angeles, Los Angeles, CA USA; 5 Department of Neurosurgery, David Geffen School of Medicine, University of California Los Angeles, Los Angeles, CA USA; 6 Department of Psychiatry and Biobehavioral Sciences, David Geffen School of Medicine, University of California Los Angeles, Los Angeles, CA USA

**Keywords:** Neuroscience, Spinal cord diseases

## Abstract

Patients with degenerative cervical myelopathy (DCM) experience structural and functional brain reorganization. However, few studies have investigated the influence of sex on cerebral alterations. The present study investigates the role of sex on brain functional connectivity (FC) and global network topology in DCM and healthy controls (HCs). The resting-state functional MRI data was acquired for 100 patients (58 males vs. 42 females). ROI-to-ROI FC and network topological features were characterized for each patient and HC. Group differences in FC and network topological features were examined. Compared to healthy counterparts, DCM males exhibited higher FC between vision-related brain regions, and cerebellum, brainstem, and thalamus, but lower FC between the intracalcarine cortex and frontal and somatosensory cortices, while DCM females demonstrated higher FC between the thalamus and cerebellar and sensorimotor regions, but lower FC between sensorimotor and visual regions. DCM males displayed higher FC within the cerebellum and between the posterior cingulate cortex (PCC) and vision-related regions, while DCM females displayed higher FC between frontal regions and the PCC, cerebellum, and visual regions. Additionally, DCM males displayed significantly greater intra-network connectivity and efficiency compared to healthy counterparts. Results from the present study imply sex-specific supraspinal functional alterations occur in patients with DCM.

## Introduction

Degenerative cervical myelopathy (DCM) is a progressive condition caused by age-related degeneration of osseocartilaginous structures within the cervical spine, resulting in chronic compression of the cervical spinal cord^1,2^. Spondylotic alterations are often initiated by spinal disc degeneration and herniation, which can lead to hypertrophy of ligamentous tissues and/or bone spurs that ultimately put strain on the spinal joints, narrow the spinal canal, and compress the cord^3^. Chronic spinal cord compression may result in neurological deficits including loss of fine motor skills and dexterity, numbness in the hands and fingers, weakness in the upper limbs, and lower limb dyscoordination^1,3^. The degree of spinal cord compression often progressively increases, resulting in irreversible damage to the cord and the gradual worsening of symptoms^4^. DCM is the most common cause of spinal cord injury in developed countries with increasing prevalence as the aging population continues to grow^4,5^.

Aside from affecting the spinal cord, chronic compression induces upstream structural and functional alterations within the brain and brainstem^6–12^. Several studies have revealed that patients with DCM experience significant reductions in cortical volume and thickness in somatosensory, motor, and pain related regions, including the somatosensory cortex, frontal gyrus, cingulate cortex, cerebellum, precuneus, and putamen when compared to healthy controls (HCs)^7,10,13^. Additionally, DCM patients exhibit altered functional connectivity (FC) in sensorimotor and pain regions, as well as in regions important to visual processing^6,8,12,13^. The degree to which morphometric and functional alterations occur has been correlated to the severity of patients’ neurological deficit and pain. Thus, it is believed that the cerebral alterations observed in DCM patients are compensatory mechanisms for the preservation of neurological function. Although these previous studies have identified unique supraspinal features associated with DCM, the influence of sex on cerebral alterations and disease pathology remains largely unexplored.

Cervical myelopathy is more common in males than females with a reported incidence ratio as high as 2.7-to-1^14^. When investigating the differences in cortical morphometry between males and females with DCM, recent results have suggested male DCM patients exhibit larger gray matter volume (GMV) in sensorimotor regions, as well as more anatomically precise associations between GMV and neurological deficit than female patients^15^. Research in traumatic spinal cord injury (SCI) offers ample support for sex-dependent differences in neurotraumatic response, which may be applied to understand DCM pathophysiology. Studies in both animal models and human patients show spinal cord gene expression to differ between males and females following traumatic SCI, with females exhibiting greater functional recovery following injury^16–18^. These findings support the hypothesis that DCM may result in sex-dependent alterations of brain structure.

In the present study, resting state functional magnetic resonance imaging (rs-fMRI) was used to investigate the influence of sex on FC in patients with DCM. We performed both a whole brain ROI-ROI analysis and a more targeted ROI analysis using a subset of 59 brain regions known from previous studies to be involved in DCM plastic changes^8,13,19–21^. Furthermore, graph-theory based brain network analyses of rs-fMRI signals was used to examine differences in topological network properties between DCM patients and HCs, as well as between male and female patients with DCM. By treating the brain as a complex network, where anatomical regions serve as nodes and functional or anatomical connections as edges or links, we theorized graph-based network approaches could provide meaningful metrics that describe the network topological organization and the efficiency of information processing^22^, as similar studies have revealed abnormal connectivity in various neurological conditions, including Alzheimer’s disease^23^, multiple sclerosis^24^, and schizophrenia^25^. In the present study, we tested the following hypotheses: 1) sex-dependent differences in functional connectivity exist in patients with DCM, specifically between the cerebellum, sensorimotor regions, and visual networks, and 2) male patients exhibit more streamlined topological network properties, while female patients exhibit more diffuse properties. We theorized that the influence of sex on FC may provide insight into individual patients’ response to chronic neurotrauma and sex-specific compensatory alterations driven by chronic spinal cord compression.

## Results

### Subject characteristics

As summarized in Table 1, the patient cohort consisted of 58 males with a mean age of 58.1 ± 11.5 years and 42 females with a mean age of 57.3 ± 11.0 years. The age of patients ranged from 31 to 81 years. No significant difference in age between male patients and female patients was observed (Wilcoxon-Mann–Whitney test, *p* = *0.6423*). Within the patient cohort, body mass index (BMI) ranged from 18.9 to 41.0 with a mean BMI of 26.2 ± 3.9 in male patients and 26.1 ± 5.0 in female patients. There was no significant difference in BMI between male and female patients (Wilcoxon-Mann–Whitney test, *p* = *0.5509*). Patient mJOA scores ranged from 10 to 18 with a mean score of 15.3 ± 2.5 for male patients and 15.8 ± 2.2 for female patients. Of the 100 patients, 8 patients were categorized with severe myelopathy (mJOA $\le$ 11), 21 patients with moderate myelopathy (12 $\le$ mJOA $\le$ 14), and 46 patients with mild myelopathy (15 $\le$ mJOA $\le$ 17)^26^, and 25 patients with asymptomatic spinal cord compression (mJOA = 18). No significant difference in mJOA score was observed between male and female patients (Wilcoxon-Mann–Whitney test, *p* = *0.4291*).

**Table 1:** Cohort demographics.

Subject Population	Number of Subject (Male/Female)	Age (Male/Female) [min,max] *p-value*	BMI (Male/Female) [min,max] *p-value*	mJOA (Male/Female) [min,max] *p-value*
DCM Patients	100 (58/42)	(58.1 ± 11.5 / 57.3 ± 11.0) [31,81] *p* = *0.6423*	(26.2 ± 3.9 / 26.1 ± 5.0) [18.9,41.0] *p* = *0.5509*	(15.3 ± 2.5 / 15.8 ± 2.2) [10, 18] *p* = *0.4291*
Healthy Controls	59 (28/31)	(50.4 ± 5.0 / 49.6 ± 3.5) [45,73] *p* = *0.5278*	(27.1 ± 4.3 / 26.3 ± 5.2) [19.2,40.7] *p* = *0.3098*	18 *

Age is provided in mean years ± the standard deviation, minimum and maximum years, and p-value of Wilcoxon-Mann–Whitney test between age of males and females. The body mass index (BMI) is provided in mean BMI ± the standard deviation with the minimum and maximum BMI. The modified Japanese Orthopedic Association (mJOA) score is provided in mean score ± the standard deviation with the minimum and maximum scores. The p-value of Wilcoxon-Mann–Whitney test between scores of males and females is reported for age, BMI, and mJOA score. * = HCs were categorized with a mJOA score of 18 due to their healthy neurological status.

The HC cohort consisted of 28 males with a mean age of 50.4 ± 5.0 years and 31 females with a mean age of 49.6 ± 3.5 years. All HC subjects had an mJOA score of 18. There was no significant difference in age between male and female HCs (Wilcoxon-Mann–Whitney test, *p* = *0.5278*). Within the HC cohort, BMI ranged from 19.2 to 40.7 with a mean BMI of 27.1 ± 4.3 in males and 26.3 ± 5.2 in females. No significant difference in BMI was observed between male and female HCs (Wilcoxon-Mann–Whitney test, *p* = *0.3098*).

### Disease-dependent differences in ROI-to-ROI functional connectivity

**Figure 1:**
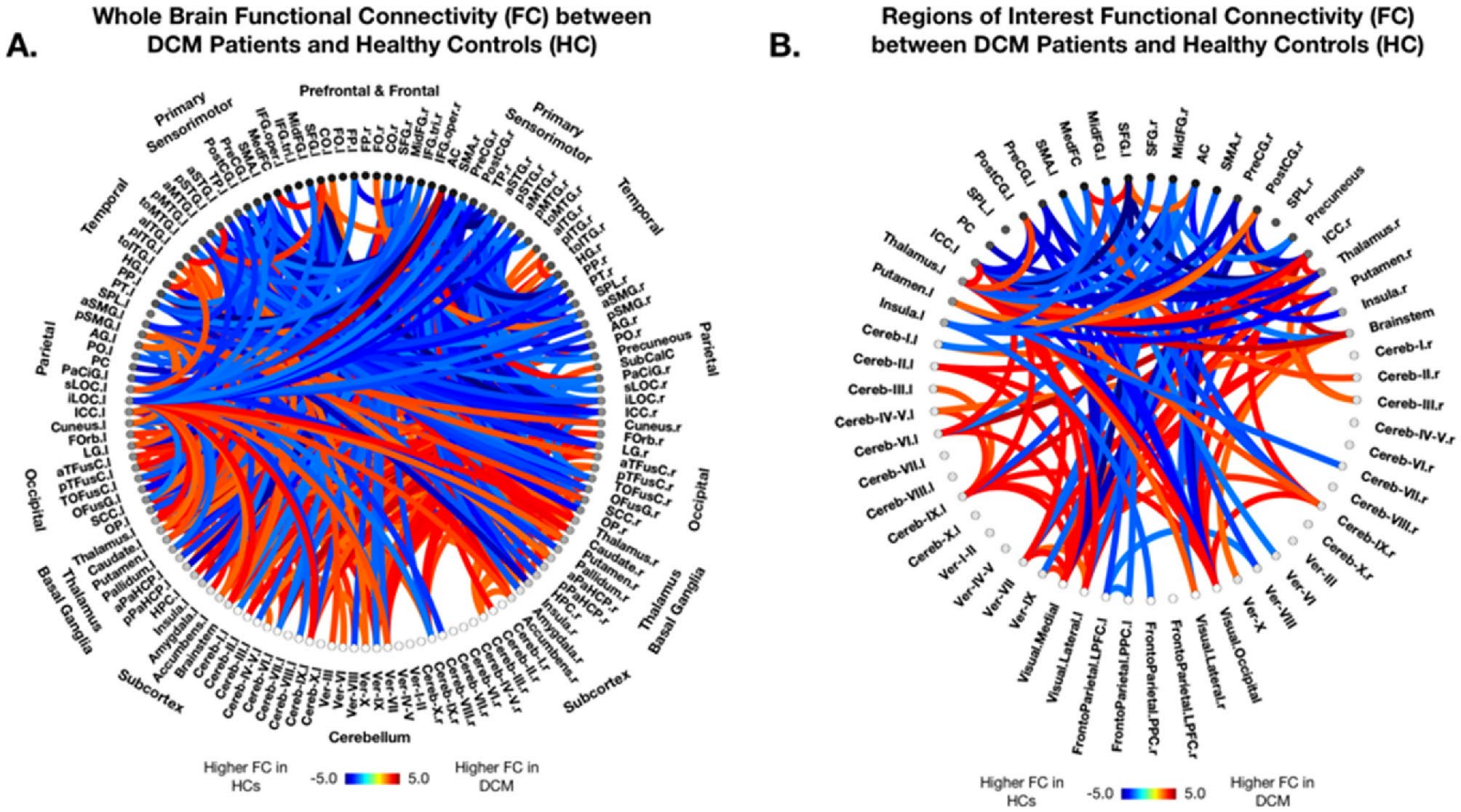
Functional Connectivity in Degenerative Cervical Myelopathy (DCM) vs Healthy Controls (HCs). ROI-to-ROI functional connectivity (FC) analysis between patients with Degenerative Cervical Myelopathy (DCM) and Healthy Controls (HCs) after regressing out the effects of age and body mass index (BMI). (**A**) Whole brain FC analysis using 132 Harvard–Oxford Automated Anatomical Labeling (AAL) atlas defined ROIs. (**B**) Regions of interest FC analysis using 59 AAL atlas defined ROIs previously shown to or hypothesized to be involved in cerebral alterations driven by DCM. Seeds of interest are listed in Table 2. (**A**–**B**) Colors denote value of T-statistic in which red–orange color denotes higher FC in DCM patients compared to HCs, while blue-light blue denotes higher FC in HCs compared to DCM patients. ROIs are labeled with l = left hemisphere and r = right hemisphere. Significant connections between ROIs were determined by thresholding based on an FDR-corrected *p*-value < 0.05.

To evaluate functional changes associated with DCM, whole-brain ROI-to-ROI functional connectivity analysis was performed between the DCM patient and HC cohorts while accounting for the effects of subject age and BMI on FC. When compared to HCs, DCM patients displayed widespread significant differences in FC (Fig. 1A). Specifically, DCM patients exhibited higher FC between primary/secondary visual ROI’s and cerebellar, thalamic, basal ganglia, and brainstem ROIs compared to HCs (Fig. 1A). Higher FC in DCM patients was also observed between thalamus and motor, limbic, and temporal ROIs, as well as between cerebellar regions. DCM patients showed lower FC compared to HCs primarily between motor regions and visual and temporal regions (Fig. 1A).

Based on previous studies identifying functional alterations in DCM patients and results from whole-brain FC analysis^8,13,19–21^, 59 ROIs were selected for further ROI-to-ROI analyses and are listed in Table 2. After subject age and BMI were regressed out, FC analysis among selected seeds of interest revealed higher FC in DCM patients compared to HCs between visual and cerebellar regions including cerebellar hemispheres and vermis, as well as between the visual networks and the brainstem, bilateral thalamus, and bilateral intracalcarine cortices (ICC) (Fig. 1B). Higher FC in DCM patients was also observed between the postcentral gyri and thalamus and putamen. In contrast, DCM patients exhibited lower FC between visual networks and motor-related brain regions including the precentral gyri, postcentral gyri, and the supplemental motor area (SMA) (Fig. 1B). Additionally, lower FC in DCM patients was observed between visual regions and frontal regions including the superior and middle frontal gyri (Fig. 1B).

**Table 2:** Regions of interest.

Regions of Interest			
Frontal		Cerebellum	
L Superior Frontal Gyrus	R Superior Frontal Gyrus	L Cerebellum Crus1	R Cerebellum Crus1
L Middle Frontal Gyrus	R Middle Frontal Gyrus	L Cerebellum Crus2	R Cerebellum Crus2
Frontal Medial Cortex		L Cerebellum 3	R Cerebellum 3
		L Cerebellum 4/5	R Cerebellum 4/5
Sensorimotor		L Cerebellum 6	R Cerebellum 6
L Precentral Gyrus	R Precentral Gyrus	L Cerebellum 7b	R Cerebellum 7b
L Postcentral Gyrs	R Postcentral Gyrs	L Cerebellum 8	R Cerebellum 8
L Supplemental Motor Area	R Supplemental Motor Area	L Cerebellum 9	R Cerebellum 9
		L Cerebellum 10	R Cerebellum 10
Parietal		Vermis 1/2	
L Superior Parietal Lobule	R Superior Parietal Lobule	Vermis 3	
L Insula	R Insula	Vermis 4/5	
Precuneus		Vermis 6	
		Vermis 7	
Thalamus/Basal Ganglia		Vermis 8	
L Thalamus	R Thalamus	Vermis 9	
L Putamen	R Putamen	Vermis 10	
Occipital Cortex		FrontoParietal Network	
L Intracalcarine Cortex	R Intracalcarine Cortex	L Lateral Prefrontal Cortex	R Lateral Prefrontal Cortex
		L Posterior Parietal Cortex	R Posterior Parietal Cortex
Cingulate Cortex			
Anterior Cingulate Gyrus	Posterior Cingulate Gyrus	Visual Network	
		L Lateral Visual Network	R Lateral Visual Network
Subcortical		Medial Visual Network	
Brainstem		Occipital Visual Network	

These Harvard–Oxford Automated Anatomical Labeling (AAL) atlas defined regions and networks were selected as regions of interest for ROI-to-ROI and ROI-to-voxel analyses.

### Sex-specific differences in ROI-to-ROI functional connectivity

**Figure 2:**
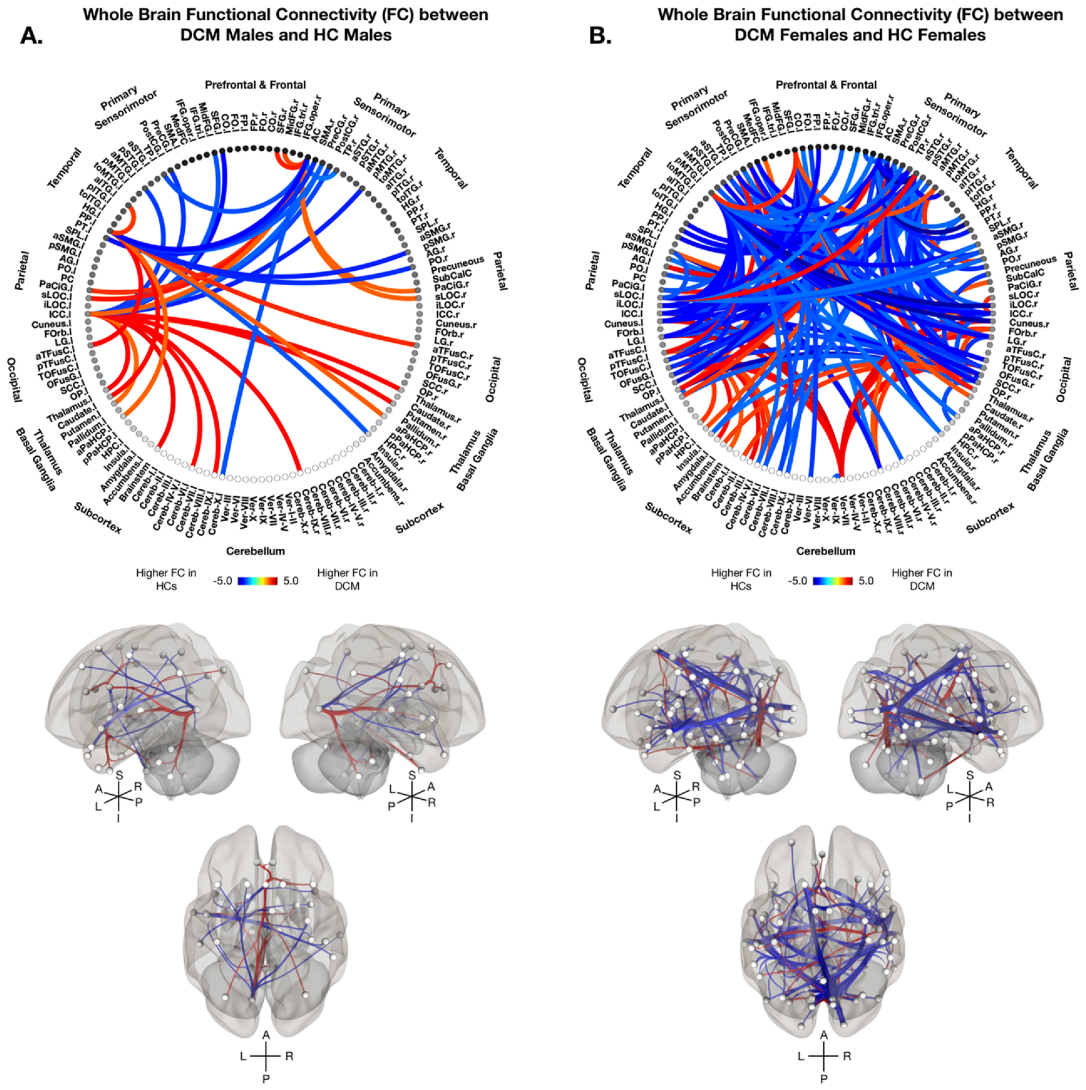
Sex-Specific Whole Brain Functional Connectivity in Degenerative Cervical Myelopathy (DCM) vs Healthy Controls (HCs). Sex-specific whole brain functional connectivity (FC) analysis using 132 Harvard–Oxford Automated Anatomical Labeling (AAL) atlas defined ROIs between (**A**) DCM males and HC males, and (**B**) DCM females and HC females after regressing out the effects of age and body mass index (BMI). (A-B, top) Colors on the functional connectome diagrams denotes the value of T-statistic in which red–orange color denotes higher FC in DCM patients compared to HCs, while blue-light blue denotes higher FC in HCs compared to DCM patients. ROIs are labeled with l = left hemisphere and r = right hemisphere. (A-B, bottom) Left, right, and superiors views of a 3D brain rendering illustrating significant ROIs as spheres and significant differences in FC between the ROIs as lines with red denoting higher FC in DCM patients compared to HCs and blue denoting higher FC in HCs compared to DCM patients. Lighter spheres represent more superficial ROIs relative to the viewer, while darker sphere represent ROIs deeper within the brain. Significant connections between ROIs were determined by thresholding based on an FDR-corrected *p*-value < 0.05.

Results from the whole-brain and select seeds of interest ROI-to-ROI analyses revealed no significant differences in FC between males and females within the HC cohort when accounting for age and BMI. In contrast, significant sex-specific differences between DCM patients and HCs were observed for both whole-brain and seeds of interest FC analyses. Whole-brain analysis between male DCM patients and male HCs revealed higher FC in DCM males between the left ICC and bilateral thalamus, bilateral caudate, brainstem, and various cerebellar regions (Fig. 2A). DCM males also displayed higher FC between the anterior cingulate cortex (AC) and bilateral parietal regions, including the superior lateral occipital cortex (sLOC) and paracingulate gyrus (PaCiG), (Fig. 2A). Compared with healthy males, DCM males exhibited lower FC between sensorimotor and temporal/parietal regions, specifically the left ICC and left bilateral planum polare (PP) (Fig. 2A).

In females, FC analysis revealed higher FC between the cerebellum, parietal, and occipital regions in DCM females compared to HC females (Fig. 2B). Specifically, higher FC was observed between the cerebellar vermis region VII and various parietal/occipital regions including bilateral ICC, bilateral cuneus, bilateral lingual gyri (LG), and bilateral fusiform cortices (FusC/FusG). Higher FC in female patients was also observed between the thalamus and the right postcentral gyrus, while lower FC was observed across sensorimotor, temporal, and occipital regions (Fig. 2B). Bilateral primary sensorimotor regions, including the SMA and the precentral and postcentral gyri, displayed lower FC with the LOC, ICC, cuneus, and fusiform cortices in DCM females as compared to HC females (Fig. 2B). Generally, male patients demonstrated fewer but more specific alterations in FC, while female patients exhibited more widespread and diffuse alterations when compared to healthy counterparts.

**Figure 3:**
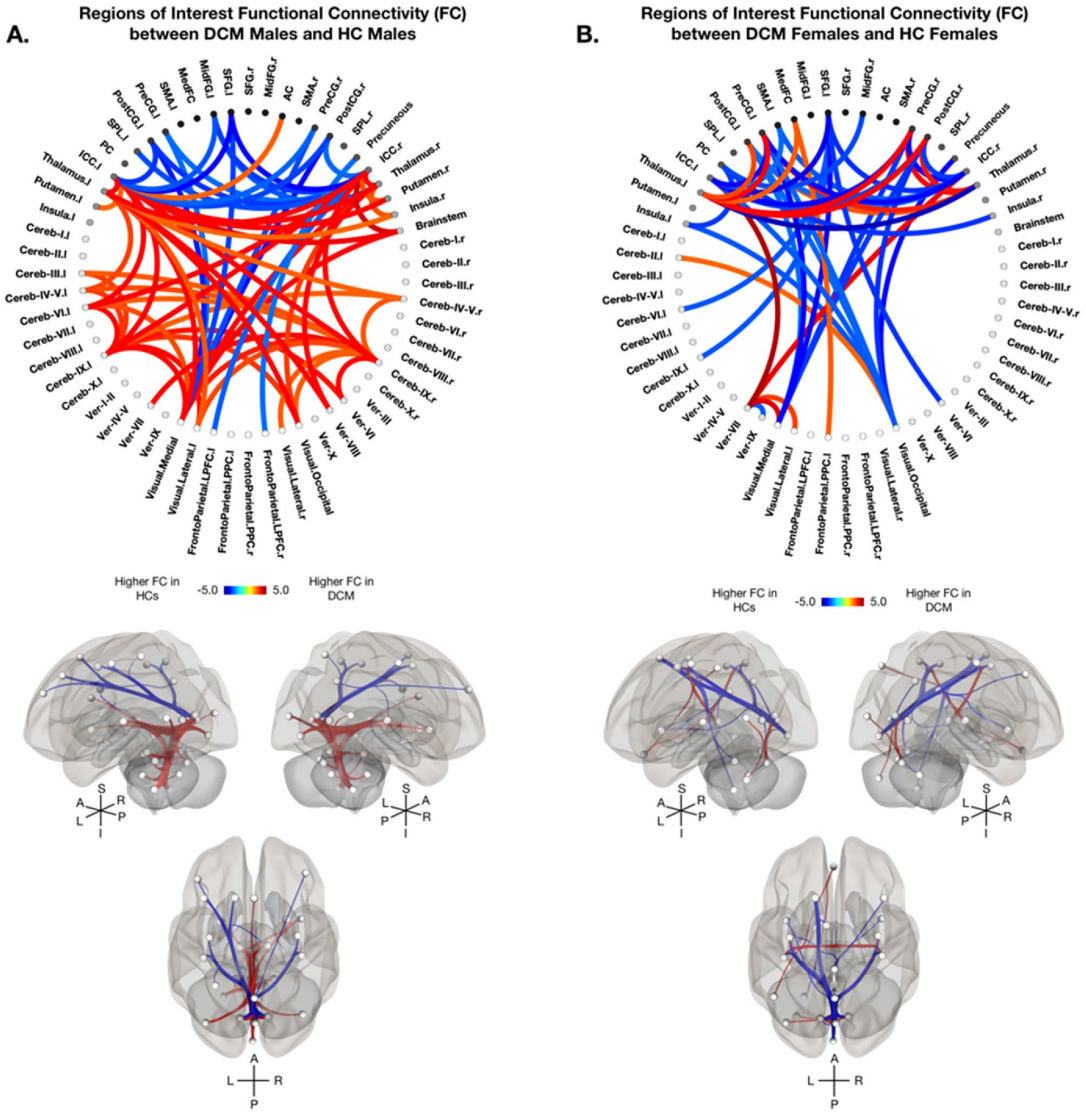
Sex-Specific Regions of Interest Functional Connectivity in Degenerative Cervical Myelopathy (DCM) vs Healthy Controls (HCs). Sex-specific regions of interest functional connectivity (FC) analysis using 59 AAL atlas defined ROIs between (**A**) DCM males and HC males, and (**B**) DCM females and HC females after regressing out the effects of age and body mass index (BMI). (A-B, top) Colors on the functional connectome diagrams denotes the value of T-statistic in which red–orange color denotes higher FC in DCM patients compared to HCs, while blue-light blue denotes higher FC in HCs compared to DCM patients. ROIs are labeled with l = left hemisphere and r = right hemisphere. (A-B, bottom) Left, right, and superiors views of a 3D brain rendering illustrating significant ROIs as spheres and significant differences in FC between the ROIs as lines with red denoting higher FC in DCM patients compared to HCs and blue denoting higher FC in HCs compared to DCM patients. Lighter spheres represent more superficial ROIs relative to the viewer, while darker sphere represent ROIs deeper within the brain. Significant connections between ROIs were determined by thresholding based on an FDR-corrected *p*-value < 0.05.

In addition to whole-brain connectivity analyses, seeds of interest ROI-to-ROI analyses were performed to provide a more refined investigation of sex-specific FC differences in DCM. When comparing DCM males and HC males, higher FC in male patients was observed primarily between cerebellar hemispheres, thalamic and visual regions (Fig. 3A). DCM males displayed higher FC between bilateral ICC and cerebellar regions, the thalamus, the putamen, and the brainstem (Fig. 3A). DCM males displayed lower FC between the intracalarine cortices and the pre- and post- central gyri, as well as between visual regions and frontal regions (Fig. 3A). In contrast, DCM females displayed higher FC between the thalamus and bilateral sensorimotor regions, as well as between the cerebellar vermis and bilateral ICC when compared to HC females (Fig. 3B). Additionally, DCM females demonstrated lower FC between the ICC and sensorimotor regions including the pre- and post- central gyri and SMA, as well as between visual, motor, and frontal regions (Fig. 3B).

**Figure 4:**
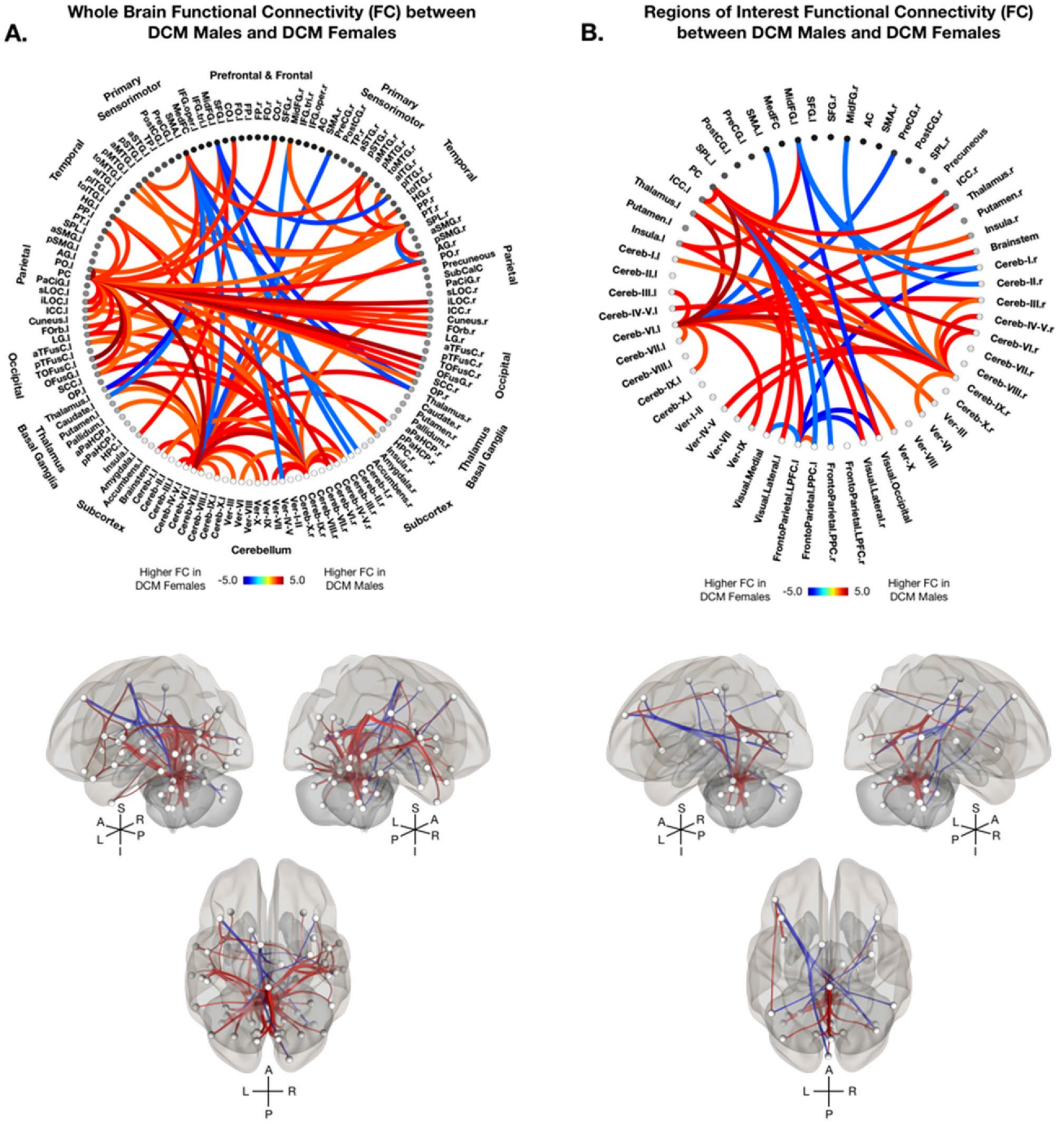
Functional Connectivity in Degenerative Cervical Myelopathy (DCM) Males vs Females. (**A**) Sex-specific whole brain functional connectivity (FC) analysis using 132 Harvard–Oxford Automated Anatomical Labeling (AAL) atlas defined ROIs between DCM males and DCM females after regressing out the effects of age and body mass index (BMI). (**B**) Regions of interest functional connectivity (FC) analysis using 59 AAL atlas defined ROIs between male and female DCM patients after regressing out the effects of age and body mass index (BMI). (A-B, top) Colors on the functional connectome diagrams denotes the value of T-statistic in which red–orange color denotes higher FC in DCM males compared to DCM females, while blue-light blue denotes higher FC in DCM females compared to DCM males. ROIs are labeled with l = left hemisphere and r = right hemisphere. (A-B, bottom) Left, right, and superiors views of a 3D brain rendering illustrating significant ROIs as spheres and significant differences in FC between the ROIs as lines with red denoting higher FC in DCM males compared to DCM females and blue denoting higher FC in DCM females compared to DCM males. Lighter spheres represent more superficial ROIs relative to the viewer, while darker sphere represent ROIs deeper within the brain. Significant connections between ROIs were determined by thresholding based on an FDR-corrected *p*-value < 0.05.

Whole-brain FC analyses revealed DCM males experience higher FC primarily within the cerebellum, between cerebellar and parietal/occipital regions compared to DCM females (Fig. 4A). DCM males also displayed higher FC between the posterior cingulate cortex and frontal, parietal, temporal, and cerebellar regions (Fig. 4A). DCM females demonstrated higher FC between sensorimotor regions and the thalamus and cerebellum compared to DCM males (Fig. 4A). Furthermore, seeds of interest analyses demonstrated significant sex-dependent differences in FC in DCM, in which higher FC was observed between within the cerebellum and between cerebellar regions and the thalamus and brainstem in DCM males compared to DCM females (Fig. 4B). DCM males also observed higher FC compared to DCM females between the posterior cingulate cortex and the ICC, the cerebellum, and visual regions (Fig. 4B). Whereas DCM females exhibited higher FC between fronto-parietal regions and the posterior cingulate cortex, cerebellum, and visual regions (Fig. 4B). Additionally, DCM females displayed higher FC between cerebellar and sensorimotor and frontal regions compared to DCM males (Fig. 4B).

### Network characterization

**Figure 5:**
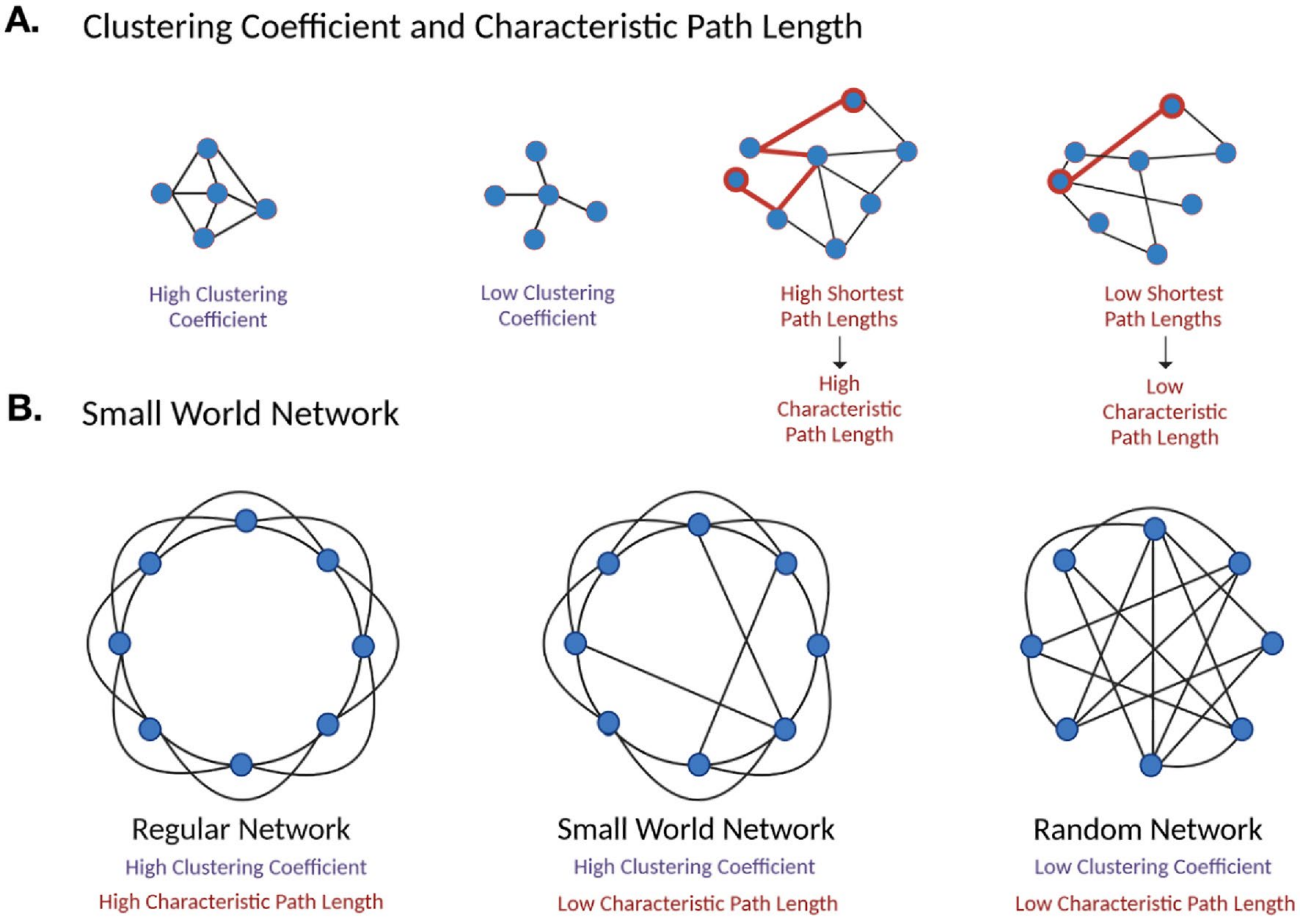
Illustration of network measurements and characterizations. (**A**) The clustering coefficient value measures the degree to which nodes in a network are clustered through their connectivity, or how well-connected a group of nodes are to one another (left). The characteristic path length is a measure of the average shortest path length between all pairs of nodes in a network (right). (**B**) A regular network possesses an ordered pattern of connectivity, resulting in a high clustering coefficient and high characteristic path length (left). A random network possesses an unordered, random pattern of connectivity, resulting in a low clustering coefficient and low characteristic path length (right). A small world network possesses an intermediate degree of regularity and randomness in its connectivity, resulting in a high clustering coefficient and low characteristic path length (middle).

**Figure 6:**
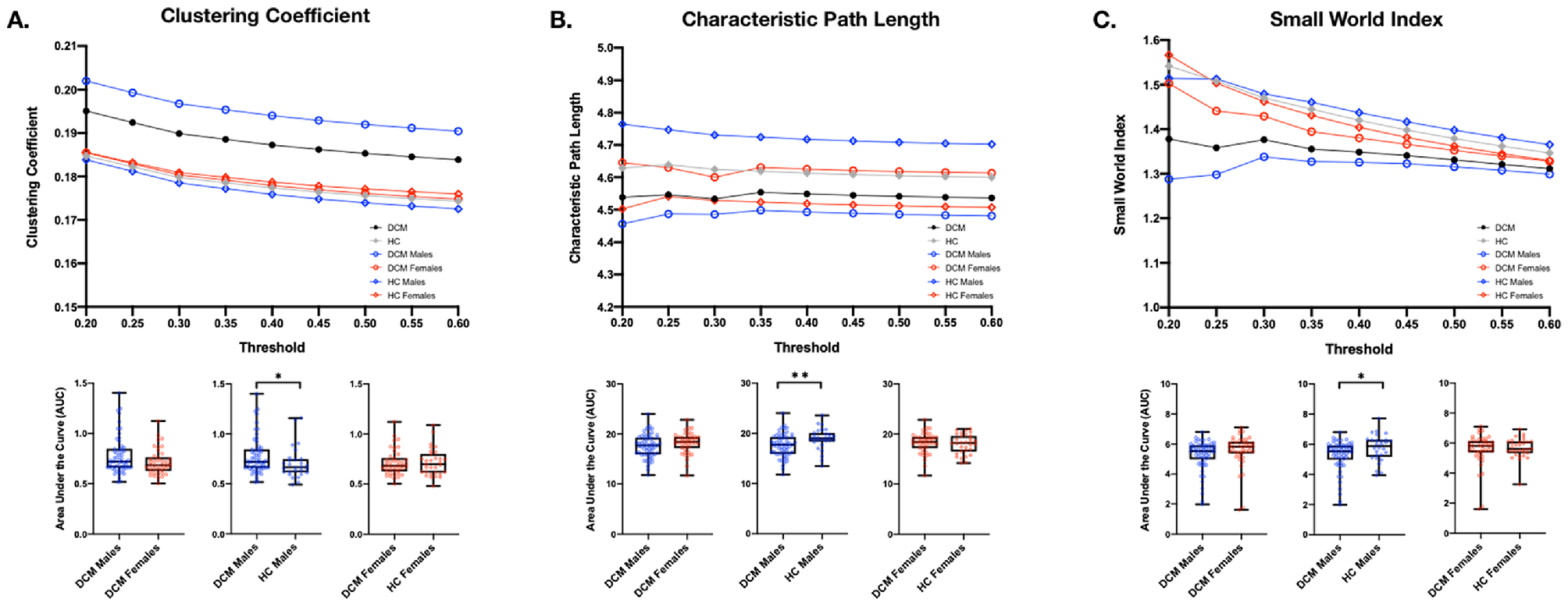
Graph Theory-based Network Properties in Degenerative Cervical Myelopathy (DCM) and Healthy Controls (HCs). Graph theory-based network measurements, including (**A**) global clustering coefficient, (**B**) characteristic path length, and (**C**) small-world index are plotted for each network threshold ranging from 0.2 to 0.6 for the DCM cohort (black circle), HC cohort (gray diamond), DCM males (blue circle), DCM females (red circle), HC males (blue diamond), and HC females (red diamond) (top row). The area under the curve (AUC) for each subject’s (**A**) global clustering coefficient, (**B**) characteristic path length, and (**C**) small-world index are plotted and compared between the following cohorts, (1) DCM males and DCM females, (2) DCM males and HC males, and (3) DCM females and HC females (bottom row). “*” and “**” refer to statistically significant difference in network measurement between cohorts of (*P* < 0.05) and (*P* < 0.01).

Graph-theory based network analyses of rs-fMRI signals were employed to investigate sex-specific differences in global network topology. The clustering coefficient, path length, and small-world index were calculated for each subject based on their respective functional connectivity matrix. As illustrated in Fig. 5, the clustering coefficient reflects general network connectivity (i.e., higher clustering coefficient, more connected network) and the path length represents efficiency of information transfer through the network (i.e., low path length, more efficient network). No statistically significant difference in area under the curve (AUC) for subjects’ clustering coefficient, characteristic path length, and small-world index were observed between the HC males and HC females, nor between DCM males and DCM females (Fig. 6). However, DCM males exhibited significantly higher clustering coefficient AUC (Fig. 6A, *P* = *0.0363*), lower characteristic path length AUC (Fig. 6B, *P* = *0.0080*), and lower small-world index AUC (Fig. 6C, *P* = *0.0317*) than HC males. No significant difference was observed in AUC between DCM females and HC females.

## Discussion

Using resting-state fMRI and graph-theory based network characterization, the present study demonstrated significant sex-specific alterations in FC in patients with DCM compared with age-matched HCs. Female DCM patients exhibited more widespread, yet subtle changes in FC, whereas male DCM patients exhibited more defined alterations within few and specific pathways. Additionally, the current study investigated FC network-based properties between patients with DCM and HCs in addition to sex-dependent differences finding that male DCM patients showed greater overall informational network connectivity compared to healthy males indicating sex-specific differences in functional connectivity, global integration, and global segregation of whole-brain networks in DCM.

Findings from whole-brain and seed-based ROI-to-ROI FC analyses between DCM patients and HCs were consistent with the present literature, including patients exhibiting higher FC between and within visual and cerebellar regions^20,27^, higher FC between the thalamus and cerebellum^19^, lower FC between frontal and visual regions^28^, lower FC between sensorimotor and thalamic^29^, and lower FC between subcortical and frontal regions^6^ compared to HCs. However, few studies have implemented graph-theory based investigation of network differences in DCM patients have found DCM patients exhibit less optimal small-world properties (i.e., high clustering coefficient and low characteristic path length) compared to HCs^30,31^.

By modeling the brain as a complex network, graph-theory based topological analyses characterize global network structure, efficiency, and functional integration and segregation^32^. This method has been employed in previous studies to investigate the influence of sex on healthy brain connectivity, as well as the influence of various brain disorders^33–36^. In the present study, the global clustering coefficient, characteristic path length, and small-world index were quantified from functional connectivity matrices to characterize network interconnectivity, efficiency of information transfer, and overall network organization, respectively. Interestingly, DCM males and HC males exhibited significant differences in clustering coefficient, characteristic path length, and small-world index implying that potential disease-specific changes may be concealed when not accounting for the influence of sex.

Additionally, no significant difference in small-world index nor clustering coefficient were observed between healthy males and females. However, previous studies with large healthy cohorts of young adults have reported higher network connectivity (i.e., clustering coefficient) in healthy females compared to males, a trend also observed in the present study but likely due to differences in cohort age and size was not statistically significant^34,37,38^. Healthy females also demonstrated overall shorter path lengths, thus exhibiting more efficient networks than healthy males. Interestingly, DCM males and females demonstrated the opposite trends in functional integration and segregation. While male HCs tend to exhibit lower connectivity (i.e., low clustering coefficient and high characteristic path length) compared with female HCs, male DCM patients demonstrate greater intra-network connectivity and efficiency (i.e., high clustering coefficient and low characteristic path length) compared to female DCM patients, suggesting that in DCM, female brain networks are comprised of numerous weak links compared to male patients, however further analyses with a large patient cohort are necessary. When compared to healthy counterparts, DCM males exhibited greater network efficiency and connectivity indicating sex-specific topological reorganization in DCM.

ROI-to-ROI functional connectivity analyses complemented topological network findings and suggest that male DCM patients exhibited more targeted and segregated FC network alterations, while female DCM patients experience more subtle and diffuse FC alterations in response to chronic spinal cord compression. Overall, both male and female DCM patients exhibited higher FC between intracalcarine cortices (ICC) and cerebellar brain regions, lower FC between the ICC and sensorimotor and frontal regions, and lower FC between the left SFG and bilateral ICC compared to healthy participants. In contrast, only DCM males displayed higher FC between the ICC and brainstem. The intracalcarine cortex (ICC) is part of the primary visual cortex and has been shown to be active during visuospatial sequence learning^39^, a function that may be important for correction of motor related symptoms caused by chronic spinal cord compression in DCM.

While male patients demonstrated higher FC between the thalamus and ICC, female patients demonstrated higher FC between the thalamus and sensorimotor cortices compared to HCs. The thalamus is an integrative brain hub that relays sensory and motor information across cortical networks^40^, and previous studies have shown altered thalamocortical and cerebellar connectivity in DCM^6^. However, the present study expands on this previous work and suggests thalamocortical recruitment may be sex-specific, in which thalamus-ICC connections may be strengthened in males, while thalamus-sensorimotor connections are strengthened in females.

The current study also suggests sex-dependent differences in cerebellar involvement, in which males employ the cerebellar hemispheres, while females employ the cerebellar vermis, in functional connections to cortical visual regions. These regions are functionally distinct, with the cerebellar vermis (spinocerebellum) coordinating motor movements, and the cerebellar hemispheres (cerebrocerebellum) being involved in motor planning and timing^41^. When compared to HCs, DCM patients exhibit increased connectivity between the cerebellum and visual cortices, and that more effective connectivity between these areas was positively correlated with better visual recovery following surgical decompression^42^. We theorize visual-cerebellar plasticity and information integration in DCM may be sex-dependent and may influence visual feedback and motor compensation.

Furthermore, when comparing DCM males and females, male patients exhibited higher connectivity between the posterior cingulate cortex (PCC) and various visual regions including the ICC, while female patients exhibited higher FC between the PCC and frontoparietal regions. The PCC receives inputs from visual and somatosensory regions and has been shown to be involved in spatial processing, learning, and memory^43,44^. With recruitment of the PCC, males with DCM may rely more on visual compensation, while females with DCM may implement cortical motor planning and execution.

We suspect sex-specific structural and functional supraspinal differences in DCM, as well as the higher incidence of cervical myelopathy in males, may be driven in part by hormonal, neuroprotective, and compensatory differences following chronic spinal cord compression. Preclinical studies of spinal cord injury (SCI) have demonstrated that androgens (testosterone) and ovarian hormones (estradiol, estrogen, and progesterone) influence neuronal loss and myelination, prevent structural atrophy, attenuate neuroinflammation, and increase motor functional recovery^45–50^. Sex steroid concentration has also been shown to affect functional recovery following SCI in humans^51–53^. Furthermore, previous studies have demonstrated that sex steroid administration, including testosterone, estradiol, and progesterone, can induce alterations in functional connectivity of the brain^54–58^. Based on such findings, it has been theorized that androgens increase FC between subcortical regions, but decrease cortical-subcortical FC, while ovarian hormones increase cortical-cortical and cortical-subcortical FC^59^. Moreover, males and females may be utilizing different compensatory mechanisms in response to disease progression and motor-related symptoms, with females strengthening thalamo-sensorimotor connections, while males strengthen thalamo-visual and thalamo-cerebellar connections.

Future studies will be able to dive deeper into these differences with a larger cohort, as the size of our cohort leaves open the possibility of important connectional differences that were missed because they did not reach statistical significance. A larger cohort would also allow a finer tuned comparison between males and females at different levels of DCM severity (e.g. different mJOA scores). Finally, another limitation of this study is that we focused solely on functional connectivity, but combining this analysis with structural connectivity could reveal further insights into how male and female neural plasticity responds to this disease.

The present study reveals significant sex-specific supraspinal functional reorganization in patients with DCM. Overall, male patients demonstrated more densely connected and efficient brain networks that exhibited stronger bilateral intracalcarine-thalamic, ICC-brainstem, and ICC-cerebrocerebellar connectivity, while females displayed brain networks that exhibited stronger thalamic-sensorimotor and ICC-spinocerebellar connectivity, but weaker visual-motor connectivity. Our findings provide unique insights into sex-specific cerebral responses to chronic spinal cord injury and may spark novel treatment strategies in DCM.

## Experimental procedures

### Patient population

A total of 100 patients were prospectively enrolled from 2015 to 2022 in a cross-sectional study including brain and spinal cord imaging as well as a neurological examination. The cohort included 75 patients with DCM and 25 with asymptomatic spinal cord compression. All patients were recruited from the UCLA outpatient neurosurgery clinic and inclusion criteria included spinal cord compression with evidence of spinal cord deformation, mass effect, and no visible cerebrospinal fluid signal around the spinal cord at the site of maximal compression on MRI. Exclusion criteria included: 1) age < 18 or > 85, 2) previous cervical spine surgery, 3) clinical or radiological evidence of stroke or other neurologic disease, 4) musculoskeletal, degenerative joint disease, or other medical cause of weakness or pain that affected use of the hands and gait, 5) cardiac pacemaker or other non-MRI compatible implant, and 6) severe claustrophobia.

The Medical IRB Committee #3 at UCLA approved this study (IRB#11-001876). All patients signed Institutional Review Board-approved consent forms and gave informed consent, and all analyses were performed in compliance with the Health Insurance Portability and Accountability Act (HIPAA). The patient cohort consisted of 58 males and 42 females ranging in age from 31 to 81 years with a mean age of 58.1 years for males and 57.3 years for females. In addition, the mean body mass index (BMI) for male patients was 26.2 and 26.1 for female patients. Previous studies have demonstrated relationships between BMI and altered functional brain connectivity^60–62^, therefore BMI was included as a covariate in the present study. The modified Japanese Orthopedic Association (mJOA) score was used as a measure of neurological function^63^. The mJOA scale ranges from 0 to 18, with lower scores representing worse neurological impairment and an mJOA score of 18 reflecting no impairment. All patients underwent brain and spinal cord imaging at UCLA. Patient demographic data was summarized in Table 1.

### Healthy control population

A total of 59 neurologically intact age-matched healthy control (HC) volunteers were included from the UCLA Center for Neurobiology of Stress and Resilience (CNSR). The HC cohort consisted of 28 males and 31 females ranging in age from 45 to 73 years with a mean age of 50.4 years for males and 49.6 years for females. The mean BMI for male HCs was 27.1 and 26.3 for female HCs. Exclusion criteria implemented by the CNSR investigators consisted of (1) significant neurological or psychiatric disorder, (2) chronic gastrointestinal disorder, (3) chronic pain disorder, (4) active autoimmune or infectious disorder, (5) history of cancer, and (6) women who are pregnant or planning to become pregnant at time of study. Healthy participant demographic data was summarized in Table 1.

### Magnetic resonance imaging acquisition

High-resolution, 1 mm isotropic, 3-dimensional T1-weighted structural MRI scans were acquired on a 3 T MR scanner (Siemens Prisma; Siemens Healthcare, Erlangen, Germany) using a magnetization-prepared rapid gradient-echo (MPRAGE) sequence in either the coronal, sagittal, or axial orientation, with a repetition time (TR) of 2300–2500 ms, a minimum echo time (TE) between 2–3 ms, an inversion time (TI) of 900–945 ms, a flip angle of 9–20°, slice thickness of 1 mm with no interslice gap, a field of view (FOV) of 240 × 320 mm and matrix size of 240 × 320.

Additionally, resting state functional MRI scans were acquired with a TR of 1500–2200 ms, TE of 28–30 ms, flip angle of 77°, FOV of 220 to 245 mm with an acquisition matrix of 64 × 64 for an in-plane resolution of 3.4 to 3.8 mm, interleaved acquisition, slice thickness of 4 mm with no interslice gap, parallel imaging via CAIPIRNHA with a factor of 2, and multi-band acceleration with a factor of 2.

### Image processing and analysis

Functional MR images were processed using the CONN toolbox (https://www.nitrc.org/projects/conn)^64^, which utilizes functions from the Statistic Parametric Mapping (SPM) toolbox (http://www.fil.ion.ucl.ac.uk/spm/). Functional and structural images were processed using the standard built-in preprocessing pipeline provided in the CONN toolbox. The default pipeline performs the following steps: (1) realignment of functional images including motion correction based on 12 degrees of freedom and unwarping, (2) slice-timing correction to correct for difference in acquisition time between slices, (3) registration of functional images to structural images, (4) registration of structural images to the Montreal Neurological Institute (MNI) defined standardized space, (5) removal of signal intensity spikes and functional volumes with excessive motion (threshold set at 2 mm translation and 2° rotation in any direction) using the Artifacts Detection Tool (ART) from SPM, (6) segmentation and normalization of functional images based on tissue type (gray matter, white matter, and cerebrospinal fluid) in MNI space, (6) segmentation and normalization of structural images based on tissue type (gray matter, white matter, and cerebrospinal fluid) in MNI space, and (7) spatial smoothing of functional data using 8 mm, full width at half maximum (FWHM) Gaussian kernel. Additionally, signals from the white matter, CSF, and motion parameters were regressed from the functional data, and signal filtering was performed using a band-pass filter of 0.008–0.10 Hz to reduce noise caused by scanner drift and physiological effects (i.e., pulsation and respiration).

Functional connectivity (FC) of the brain was assessed using (1) whole brain ROI-to-ROI (also termed seed-to-seed) and (2) seeds of interest ROI-to-ROI analyses. Regions of interest (ROIs) were selected from the Harvard–Oxford Automated Anatomical Labeling (AAL) atlases^65^. Whole-brain ROI-to-ROI analyses used 132 cerebral ROIs, while seeds of interest were selected based on previously published studies which observed altered FC between DCM patients and HCs^8,13,19–21^. For the seeds of interest ROI-to-ROI analyses, the following ROIs were selected: the superior frontal gyrus (sFG), the middle frontal gyrus (mFG), the frontal medial cortex, the precentral gyrus, the postcentral gyrus, the supplementary motor area (SMA), the superior parietal lobule (SPL), the insular cortex, the precuneus, the thalamus, the putamen, intracalcarine cortex (ICC), the anterior and posterior cingulate gyri, the brainstem, the cerebellar crus (1 and 2), the cerebellar hemispheres (3, 4/5, 6, 7b, 8, 9, and 10), the cerebellar vermis (1/2,3,4/5,6,7,8,9, and 10), the lateral prefrontal cortex, the posterior parietal cortex, and the lateral, medial, and occipital visual networks (Table 2). General linear models (GLMs) were used to evaluate group differences in functional connectivity while accounting for the effects of subject age and BMI. Statistical analyses were performed using the CONN toolbox^64^, MATLAB (Release 2018a, MathWorks, Natick, MA), and GraphPad Prism software (Version 7.0c GraphPad Software, San Diego, California). For the connection threshold, significance was set at* p* < 0.01 and at the ROI level, significance was set at a false discovery rate (FDR) corrected *p* < 0.05.

### Graph theory-based network connectivity analyses

A graph theory-based approach was used to model the brain as a complex network and investigate its topological organization. This network is represented as a graph consisting of anatomical nodes and their pairwise connections or edges^66^. Here, nodes were defined as 132 anatomical ROIs derived from the Harvard–Oxford Automated Anatomical Labeling (AAL) atlases^65^. The nodes used in this analysis were consistent with the regions used in the whole-brain functional connectivity analyses. In the present study, edges were extracted from rs-fMRI data and defined as functional connectivity correlations between ROIs. The connectivity matrix (connectivity values between nodes) of each subject was calculated using the CONN toolbox^64^. In this network, the edges were weighted and undirected. Measures of global (network-wise) connectivity properties were calculated and analyzed using Matlab and the Graph Theory GLM (GTG) toolbox (www.nitrc.org/projects/metalab_gtg). In the present study, the clustering coefficient, characteristic path length, and small-world index were calculated for each subject. Graph theory-based measures are illustrated in Fig. 5. Because connectivity values vary between subjects due to noise and intrasubject variation, multiple thresholds were applied to subject’s connectivity matrices^66^. Thresholds ranged from 0.2 to 0.6 with a step of 0.05 (i.e., 0.2 refers to 20 percent of the strongest connections are maintained). Clustering coefficient, characteristic path length, and small-world index were calculated for the above threshold range and plotted using GraphPad Prism (Version 7.0c GraphPad Software, San Diego, California). The area under the curve (AUC) was calculated for each cohort and compared between groups using the Mann–Whitney unpaired t-test on GraphPad Prism. Level of significance was set at *p* < 0.05.

### Node degree

Node degree (*k*) is a local (nodal) connectivity measurement defined as the number of edges or connections maintained by each node (*i*) and is calculated as

$${k}_{i}=\sum_{j\ne i}{a}_{ij}$$

in which a_ij_ is the *i*th row and the *j*th column of the connectivity matrix^22^. The more connections a node has (higher node degree), the more important that node (i.e., anatomical brain region) may be in the brain network^32^. In addition, node degree represents how connected a node is with the rest of the nodes in a brain network^22^.

### Clustering coefficient

For each node (*i*), the clustering coefficient (*C*) measures the proportion of neighboring nodes that are interconnected (i.e., the number of triangles in the network)^67^. The clustering coefficient is a local (nodal) measurement, while the average clustering coefficient acts as a global (network) measurement^67^. The local clustering coefficient is calculated by dividing the number of connected edges between node *i*’s adjacent nodes ${t}_{i}=\frac{1}{2}\sum_{i\leftrightarrow j\leftrightarrow h}{a}_{ij}{a}_{ih}{a}_{jh}$ by the number of all possible edges^32^.

$$C(i)=\frac{2{t}_{i}}{{k}_{i}({k}_{i}-1)}$$

The clustering coefficient is a measure of functional segregation and local connectedness^67^. The higher the clustering coefficient, the more densely interconnected the brain regions are^32^. By averaging the clustering coefficient of all the nodes in a network, we can measure the interconnectedness of the whole network.

$$C=\frac{1}{N}\sum_{i}C(i)=\frac{1}{N}\sum_{i}\frac{2{t}_{i}}{{k}_{i}({k}_{i}-1)}$$

### The characteristic path length

The characteristic path length (*L*) refers to the average distance or minimum number of edges between nodes in the network^22^. The shortest path length (*l*) between each node pair is measured as

$${l}_{ij}=\sum_{{a}_{st}\in {l}_{i\leftrightarrow j}}{a}_{st}$$

where ${l}_{i\leftrightarrow j}$ is the shortest path between node $i$ and node $j$. The characteristic path length (*L*) is the average of the shortest path length for all nodes within a network and is denoted as

$$L=\frac{1}{N}\sum_{i}{l}_{i}$$

Path length is a measure of functional integration and is used to characterize the internal structure of the brain network. Shorter path lengths can transmit information more rapidly and efficiently^32^. Therefore, the smaller the characteristic path length, the higher the routing efficiency of the network^22^.

### Small world brain connectivity

A small-world network is defined as having high local clustering (i.e., high clustering coefficient) and a low minimum path length between pairs of nodes (i.e., low characteristic path length)^22^. The human brain exhibits small-world network properties, with its highly connected and modular structure being ideal for efficient information processing^22^. To determine if a network is considered a small-world network, the global clustering coefficient (C) and characteristic path length (*L*) are compared with those of a random network^32^. Using the Graph Theory GLM toolbox, a null model (random network) was generated using subject data over 1000 repetitions.

$$\gamma =\frac{C}{{C}_{rand}} \lambda =\frac{L}{{L}_{rand}}$$

The following two conditions must be true:  $\gamma \gg 1$ and $\lambda \approx 1$^32^. The small-world index ($\sigma$) of a network is quantified as

$$\sigma =\frac{\gamma }{\lambda }$$

## Institutional review board

The Medical IRB Committee #3 at UCLA approved this study (IRB#11-001876).

## Informed consent

All patients signed Institutional Review Board-approved consent forms and gave informed consent, and all analyses were performed in compliance with the Health Insurance Portability and Accountability Act (HIPAA).

## Data Availability

Datasets used in the current study are available from the corresponding author on reasonable request.

## Abbreviations

DCM: Degenerative cervical myelopathy
rs-fMRI: Resting-state functional magnetic resonance imaging
FC: Functional connectivity
mJOA: Modified Japanese orthopedic association
ROI: Region of interest
FDR: False discovery rate
